# Intraspecific variability in *Phaeocystis antarctica's* response to iron and light stress

**DOI:** 10.1371/journal.pone.0179751

**Published:** 2017-07-10

**Authors:** Katja E. Luxem, Michael J. Ellwood, Robert F. Strzepek

**Affiliations:** 1 Division of Chemistry and Chemical Engineering, California Institute of Technology, Pasadena, California, United States of America; 2 Research School of Earth Sciences, Australian National University, Canberra, ACT, Australia; Beijing Normal University, CHINA

## Abstract

*Phaeocystis antarctica* is an abundant phytoplankton species in the Southern Ocean, where growth is frequently limited by iron and light. Being able to grow under low iron conditions is essential to the species’ success, but there have been hints that this ability differs among clones. Here, we compare the growth, cell size and chlorophyll *a* concentrations of four *P*. *antarctica* clones cultured under different iron and light conditions. Iron was provided either as unchelated iron (Fe′) or bound to the bacterial siderophore desferrioxamine B, representing, respectively, the most and least bioavailable forms of iron which phytoplankton encounter in the marine environment. The growth rate data demonstrate that the clones vary in their ability to grow using organically bound iron, and that this ability is not related to their ability to grow at low inorganic iron concentrations. These results are consistent at low and high light. Physiologically, only three of the four clones shrink or decrease the concentration of chlorophyll *a* in response to iron limitation, and only one clone decreases colony formation. Together, our data show that *P*. *antarctica* clones 1) respond to the same degree of iron limitation using different acclimation strategies, and 2) vary in their ability to grow under the same external iron and light conditions. This physiological diversity is surprisingly large for isolates of a single phytoplankton species.

## Introduction

*Phaeocystis antarctica* is an abundant bloom-forming phytoplankton species in the Southern Ocean, where it plays a pivotal role in the marine carbon and sulfur cycles [1, 2]. Its ability to grow under the variable, but often limiting, iron and light conditions in the Southern Ocean [3] is fundamental to its success. Some of the acclimation strategies that enable *P*. *antarctica* to thrive under these conditions include a complex life cycle with unicellular and colonial cell types [4], multiple iron uptake pathways [5] and a strict regulation of the iron containing proteins involved in photosynthesis [6, 7].

As a species, *P*. *antarctica* thrives in the low iron waters of the Southern Ocean, but there have been hints that individual *P*. *antarctica* clones may respond differently to the same external iron conditions. For example, in two laboratories studying different *P*. *antarctica* clones, a specific low iron treatment dramatically slowed growth in one clone but not in the other [5, 7]. In this project, we systematically grew three *P*. *antarctica* clones under different iron and light conditions and monitored their growth rates, cell size, chlorophyll *a* concentrations and colony formation. These results were compared with those of a fourth clone described previously [5, 6].

The clones were grown with varying concentrations of two different iron chelating ligands, ethylene diamine tetraacetic acid (EDTA) and the bacterial siderophore desferrioxamine B (DFB). The bioavailable iron species in these media, unchelated iron (Fe′) and DFB-bound iron, are thought to represent ‘end-member’ cases for the types of bioavailable iron which phytoplankton may encounter in the environment [8]. In our experiments, iron limitation was induced by increasing the ligand concentration and thereby decreasing the concentration of Fe′. We tested whether the four *P*. *antarctica* clones grew similarly under each external iron and light treatment and whether the clones used the same physiological strategies to acclimate to iron limitation.

## Materials and methods

### Experimental overview

In our experiments, three *P*. *antarctica* clones were acclimated to growth media with different concentrations of EDTA and DFB, two iron binding ligands whose chemistry within the artificial growth medium is well studied [9, 10]. Previously published data from clone AA1 collected by our research team in the past are presented for comparison [5, 6] and we chose to use the same trace metal and ligand conditions as before. All experiments were performed at low (25 μmol photons m^-2^ s^-1^) and high (200 μmol photons m^-2^ s^-1^) light levels.

In this report, the different iron treatments are referred to as iron replete (9.95 nmol L^-1^ Fe; 10 μmol L^-1^ EDTA), high EDTA (1.8 nmol L^-1^ Fe; 100 μmol L^-1^ EDTA) and high DFB (3.8 nmol L^-1^ Fe; 10 μmol L^-1^ EDTA; 400 nmol L^-1^ DFB). After acclimation to these iron limiting treatments, an iron limiting treatment which roughly halved the optimal growth rate (suggesting similar physiological extents of iron limitation) was chosen for each clone. The chosen treatments were high EDTA for clone RS24, high DFB for clones AA1 and SX9, and 3.8 nmol L^-1^ Fe, 40 nmol L^-1^ DFB for clone W51. We measured intracellular chlorophyll *a* concentrations, cell size distributions, and colony formation for these treatments at mid-exponential growth and compared them to the iron-replete values.

### Study organisms

*Phaeocystis antarctica* clones W51 and RS24 were isolated from the Ross Sea by D. Caron and provided by D. Hutchins. Clone W51 was isolated in January 1999 and clone RS24 was isolated in January 2005. Clones AA1 and SX9 were isolated by R. Strzepek in December 2001 (61°20.89' S, 139°50.69' E) and December 2004 (65°08.72' S 174°08.94' E), respectively [5]. All cultures were maintained under axenic conditions. The data for clone AA1 are taken from studies published previously [5, 6].

### Medium preparation

All phytoplankton cultures were grown in the artificial seawater medium Aquil [9] enriched with 10 μmol L^-1^ phosphate, 300 μmol L^-1^ nitrate, and filter sterilized (0.2 μm Gelman Acrodisc PF) trace metal and vitamin solutions as described previously [11]. Free trace metal ion concentrations, in the presence of 10 μmol L^-1^ EDTA as the chelating agent, were as follows (-log free-metal ion concentration = pMetal): pCu 14.07, pMn 8.17, pZn 10.79, and pCo 11.09. In the presence of 100 μmol L^-1^ EDTA as the chelating agent, free trace metal ion concentrations were: pCu 14.96, pMn 8.99, pZn 11.82, and pCo 12.17. These concentrations were calculated using the chemical equilibrium computer program Visual MINTEQ (http://www.lwr.kth.se/English/OurSoftware/vminteq/) for Aquil media at 3˚C, salinity 35, and the initial pH of the medium reported previously (8.17 ± 0.04) [5]. Selenite and molybdate were added at 10^−8^ and 10^−7^ mol L^-1^, respectively.

### Iron manipulation experiments

The *P*. *antarctica* cultures were grown in a variety of media that varied in their total Fe concentration and in the ligand used to complex Fe. The 1.8 nmol L^-1^ Fe contamination, measured previously using electrochemical techniques, was included in the calculations of total iron [5]. Iron-replete media were prepared by adding premixed, filter-sterilized FeEDTA (1:1.05) to Aquil containing 10 μmol L^-1^ EDTA for a final iron concentration of 9.95 nmol L^-1^. Inorganic iron concentrations ([Fe′]) were calculated for FeEDTA media as described previously, accounting for variances in the light field for various experiments [5].

Cultures were also grown in media where the terrestrial siderophore desferrioxamine B mesylate (DFB; Sigma-Aldrich) controlled the iron chemistry. This addition was optimized previously [5]. Briefly, DFB was precomplexed to 2 nmol L^-1^ FeCl_3_ (in 0.01 M quartz-distilled HCl) and added to Aquil medium containing 10 μmol L^-1^ EDTA to buffer the other trace metals. The clones were acclimated to growth media containing Fe:DFB molar ratios of 3.8:40, 3.8:200, and 3.8:400 nmol L^-1^. Unchelated iron concentrations (Fe′) in FeDFB media were calculated by the equation [Fe′] = [FeDFB] / [L’] x K_Fe’Lcond_ (K_Fe’Lcond_ = 10^11.8^ measured in Aquil at pH 8) [10]. A stock DFB solution (1.52 x 10^−2^ mol L^-1^) was prepared as previously described [12]. Hydroxamate siderophores with similar functional groups as DFB have been isolated from seawater [13–16] and both EDTA and DFB are commonly used in phytoplankton culturing experiments to control iron bioavailability and induce iron limitation ([9], [8] and references therein).

*P*. *antarctica* cultures were grown at 3° ± 1°C under a continuous photon flux density (PFD, μmol photons m^-2^ s^-1^) in a Percival low temperature incubator (LT-36VL, Percival, USA) with Philips Alto II fluorescent lights. Because of the complex physiological relationship between iron and light for phytoplankton from the Southern Ocean [17, 18], we grew the clones under both light limiting and light saturated conditions. The light limiting treatment was 25 μmol photons m^-2^ s^-1^ and the light replete treatment was 200 μmol photons m^-2^ s^-1^. Previous work with *P*. *antarctica* clones AA1 and SX9 suggests that growth at our low light condition (25 PFD) is light limited and growth at our high light condition (200 PFD) is light saturated [6]. PFDs were measured with a calibrated 4π quantum meter (model QSL 2101; Biospherical Instruments). It is important to be aware that the Fe-EDTA complex undergoes photochemical reactions [19] so that the iron bioavailability in the high EDTA treatment is ~3.5-fold greater in the high light condition compared to the low light condition (as shown in the Results and Discussion, Table 1). In contrast to EDTA, DFB is not photolabile and iron bioavailability is the same in the high DFB treatments at low and high light.

**Table 1:** Growth rates of *P*. *antarctica* clones under different combinations of iron, EDTA and DFB.

Iron Treatment	PFD	[Fe'] (pmol L^-1^)	n	μ (d^-1^)	μ:μmax
*Phaeocystis antarctica* (clone SX9)					
9.95 nM Fe, 10 μM EDTA	25	308.	10	0.30 (0.01)	1.00
1.8 nM Fe, 100 μM EDTA	25	5.	7	0.24 (0.02)	0.81
3.8 nM Fe, 400 nM DFB	25	0.02	9	0.20 (0.02)	0.68
9.95 nM Fe, 10 μM EDTA	200	1,066.	6	0.50 (0.03)	1.00
1.8 nM Fe, 100 μM EDTA	200	19.	7	0.34 (0.04)	0.69
3.8 nM Fe, 400 nM DFB	200	0.02	3	0.28 (0.01)	0.57
*Phaeocystis antarctica* (clone W51)					
9.95 nM Fe, 10 μM EDTA	25	308.	5	0.27 (0.01)	1.00
1.8 nM Fe, 100 μM EDTA	25	5.	5	0.23 (0.02)	0.87
3.8 nM Fe, 40 nM DFB	25	0.17	3	0.15 (0.01)	0.58
3.8 nM Fe, 200 nM DFB	25	0.03	3	ng	-
3.8 nM Fe, 400 nM DFB	25	0.02	3	ng	-
9.95 nM Fe, 10 μM EDTA	200	1,066.	4	0.29 (0.01)	1.00
1.8 nM Fe, 100 μM EDTA	200	19.	5	0.29 (0.02)	0.97
3.8 nM Fe, 40 nM DFB	200	0.17	2	0.04 (0.03)	0.13
3.8 nM Fe, 200 nM DFB	200	0.03	3	ng	-
3.8 nM Fe, 400 nM DFB	200	0.02	4	ng	-
*Phaeocystis antarctica* (clone RS24)					
9.95 nM Fe, 10 μM EDTA	25	308.	4	0.41 (0.01)	1.00
1.8 nM Fe, 100 μM EDTA	25	5.	3	0.15 (0.01)	0.37
3.8 nM Fe, 400 nM DFB	25	0.02	8	0.14 (0.01)	0.35
9.95 nM Fe, 10 μM EDTA	200	1,066.	6	0.69 (0.01)	1.00
1.8 nM Fe, 100 μM EDTA	200	19.	6	0.44 (0.03)	0.64
3.8 nM Fe, 400 nM DFB	200	0.02	6	0.41 (0.04)	0.59
*Phaeocystis antarctica* (clone AA1)					
4.4 nM Fe, 10 μM EDTA	30	145.	9	0.45 (0.01)	1.00
3.8 nM Fe, 400 nM DFB	30	0.02	9	0.15 (0.00)	0.33
4.4 nM Fe, 10 μM EDTA	90	258.	36	0.52 (0.02)	1.00
1.8 nM Fe, 100 μM EDTA	90	10.6	6	0.47 (0.00)	0.90
3.8 nM Fe, 400 nM DFB	90	0.02	39	0.28 (0.04)	0.54

This table shows the specific growth rates of the four *P*. *antarctica* clones (grown in 28 mL polycarbonate tubes) as a function of iron and light availability (PFD = photon flux density, μmol photons m^-2^ s^-1^). Standard errors are shown in parentheses. The data from clone AA1 have been published previously (Strzepek *et al*. 2011 [PFD 90], Strzepek *et al*. 2012 [PFD 30]).

Experimental cultures were inoculated using stock cultures grown in Aquil medium containing 2.2 nmol L^-1^ Fe and 10 μmol L^-1^ EDTA at < 10 μmol photons m^-2^ s^-1^ under continuous illumination. Experimental cultures were grown in triplicate and acclimated to growth conditions in 28 mL polycarbonate centrifuge tubes (Thermo Scientific Nalgene) for at least three transfers (~20 generations) before being grown in 1 L polycarbonate bottles (Thermo Scientific Nalgene) for measurements of chlorophyll *a* concentrations, cell size and colony formation. Acclimated cultures were mixed daily by hand and maintained in exponential phase by dilution. Growth rates of acclimated cells in the 28 mL tubes were determined from *in vivo* chlorophyll *a* fluorescence using a Turner Designs model 10-AU fluorometer. Specific growth rates (d^-1^) were calculated from least-squares regressions of ln (*in vivo* fluorescence) vs. time during the exponential growth phase.

### Cell size and cell counts

Cell sizes were measured using a Multisizer 4 Coulter Counter (Beckman Coulter) using a spherical approximation for the *P*. *antarctica* cells. Samples were measured directly after addition to an isohaline solution, and these data were used to analyze cell size and single cell counts. Glutaraldehyde (Sigma Aldrich) was observed by visual microscopy to break up the colonies at a final concentration of 2.2% (v/v). It was added to samples that were shaken vigorously for 30 seconds and measured directly. These data were used to obtain the total (single and colonial) cell counts. The data were analyzed using the Beckman Coulter Multisizer v4.01 software. Diameter, surface area, and cell volume were analyzed separately to account for deviations from the normal distribution. As reported previously for other species [20], glutaraldehyde changed cell size inconsistently among clones and treatments, and thus the sizes of colonial cells are not reported.

### Chlorophyll *a* samples

After an acclimation period of at least three transfers (where cells were grown to mid-exponential in 28 mL polycarbonate centrifuge tubes and re-inoculated) the *P*. *antarctica* clones were grown in 1 L polycarbonate bottles in triplicate. The cells were harvested at mid-exponential growth phase for measurement of total chlorophyll *a*. Chlorophyll *a* samples were collected by gently filtering 20 mL of cell containing media onto 25 mm Type A/E Glass Fiber Filters (Pall Corporation) and rinsing with synthetic ocean water (SOW). Samples were stored in the dark at -20°C, extracted in 90% acetone in the dark for <24 hours at -20°C, and analyzed by *in vivo* fluorometry on a Turner Designs model 10-AU fluorometer calibrated with spectrophotometrically measured spinach chlorophyll *a* standards (Sigma Aldrich). Cell counts and cell volumes of unpreserved cells were measured at harvest, and the chlorophyll *a* measurements were normalized to total cell counts. Intracellular chlorophyll *a* concentrations were calculated using the cell volume of free living cells as an approximation.

### Data analysis

Sample means, standard deviations, and fold-change were calculated using Microsoft Excel software (version 2010). Analysis of Variance (ANOVA) was used to determine treatment effects and significant results are reported at the 95 percent confidence level (p < 0.05).

## Results and discussion

### Growth in iron limiting media differs among clones

**Fig 1:**
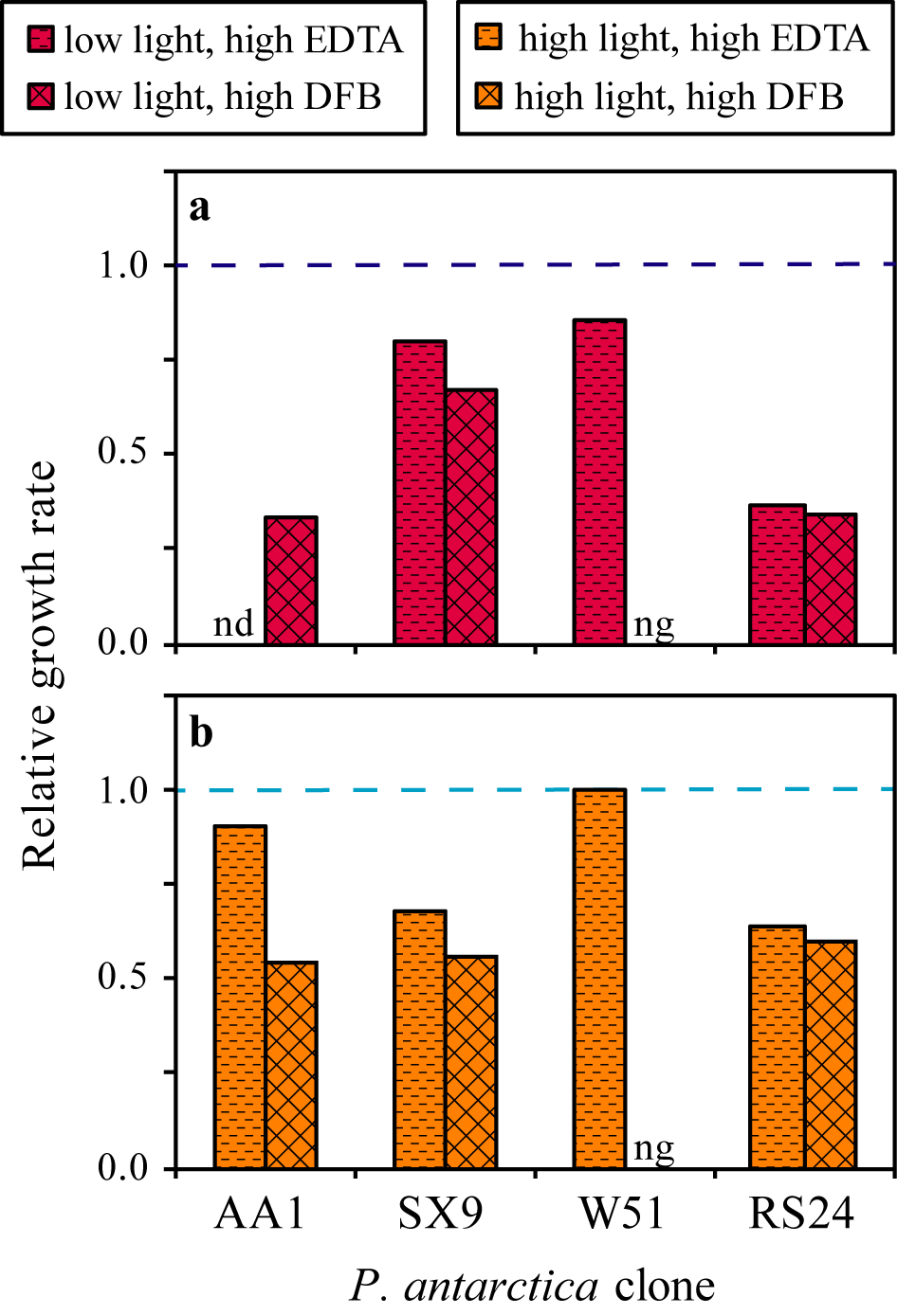
Extent of iron limitation in the high EDTA and DFB media differs among clones. The relative growth rates are the growth rates of the clones in the high EDTA or high DFB treatment divided by their growth rates in the iron replete treatment at low (a) and high (b) light. The dashed line (relative growth rate = 1) represents the normalized growth rate of each clone in the iron replete medium. For clone W51, “ng” (no growth) indicates that the clone did not grow in the high DFB medium. For clone AA1, “nd” (no data) were collected for the high EDTA medium at low light. This figure shows that, among the four clones, the extent of iron limitation under growth at low Fe′ is not related to the extent of iron limitation under growth on DFB-bound iron. This is particularly evident when comparing clone W51, which is iron replete in the high EDTA medium but does not grow in the high DFB medium, to the other clones. The data from clone AA1 have been published previously (Strzepek *et al*. 2011 [high light], Strzepek *et al*. 2012 [low light]).

**Fig 2:**
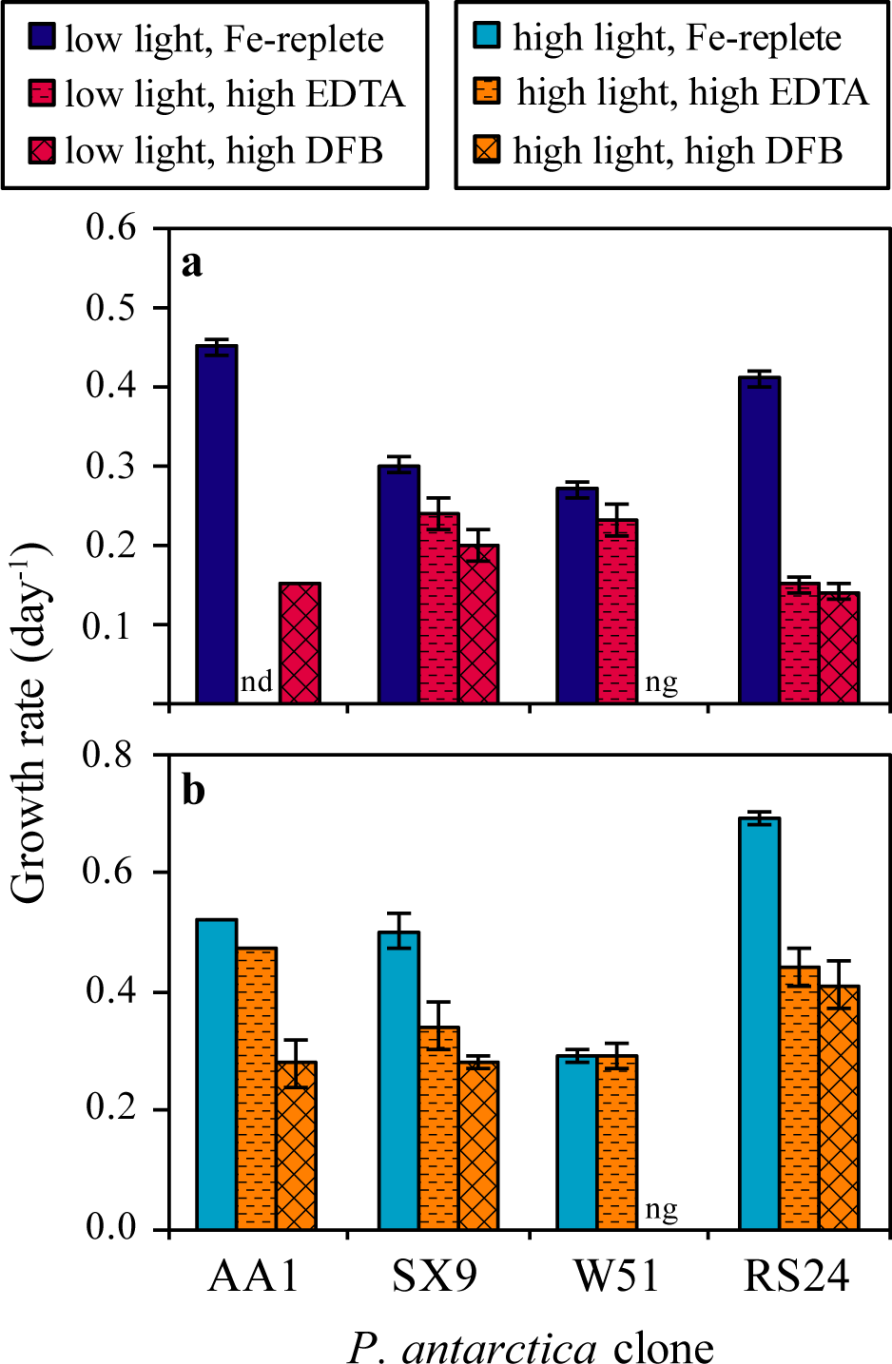
Absolute growth rates of *P*. *antarctica* clones under various low iron conditions. These graphs show the absolute growth rates (day^-1^) of the four *P*. *antarctica* clones grown in iron replete, high EDTA and high DFB media under low (a) and high (b) light conditions. For clone W51, “ng” (no growth) indicates that the clone did not grow in the high DFB medium. For clone AA1, “nd” (no data) were collected in the high EDTA medium at low light. The error bars represent the standard error of the mean of the growth rate (n is provided for each treatment in Table 1). The data from clone AA1 have been published previously (Strzepek *et al*. 2011 [high light], Strzepek *et al*. 2012 [low light]).

The *P*. *antarctica* clones were grown in two low iron media, high EDTA and high DFB (Fig 1, Table 1). To account for the wide range in the clones’ iron replete growth rates, we compare the iron limitation experienced by each clone relative to its iron replete growth rate (Fig 2). Clones SX9, AA1 and W51 grow more slowly in the high DFB medium than in the high EDTA medium. Indeed, clone W51 does not grow at all in the high DFB medium until the concentration of DFB is decreased by an order of magnitude. Conversely, in the high EDTA medium, clone W51 experiences the lowest degree of iron limitation, followed by clones AA1 and SX9. Clone AA1 grows relatively well in both the high DFB and high EDTA media. Among the clones, there is no clear relationship between the extent of iron limitation when grown under low concentrations of Fe′ (high EDTA medium) or with DFB-bound iron (high DFB medium). These growth rate patterns are consistent under both light conditions and show that *P*. *antarctica* clones have varying abilities to grow under the same external iron conditions.

### Clone W51 grows in high EDTA but not high DFB media

**Fig 3:**
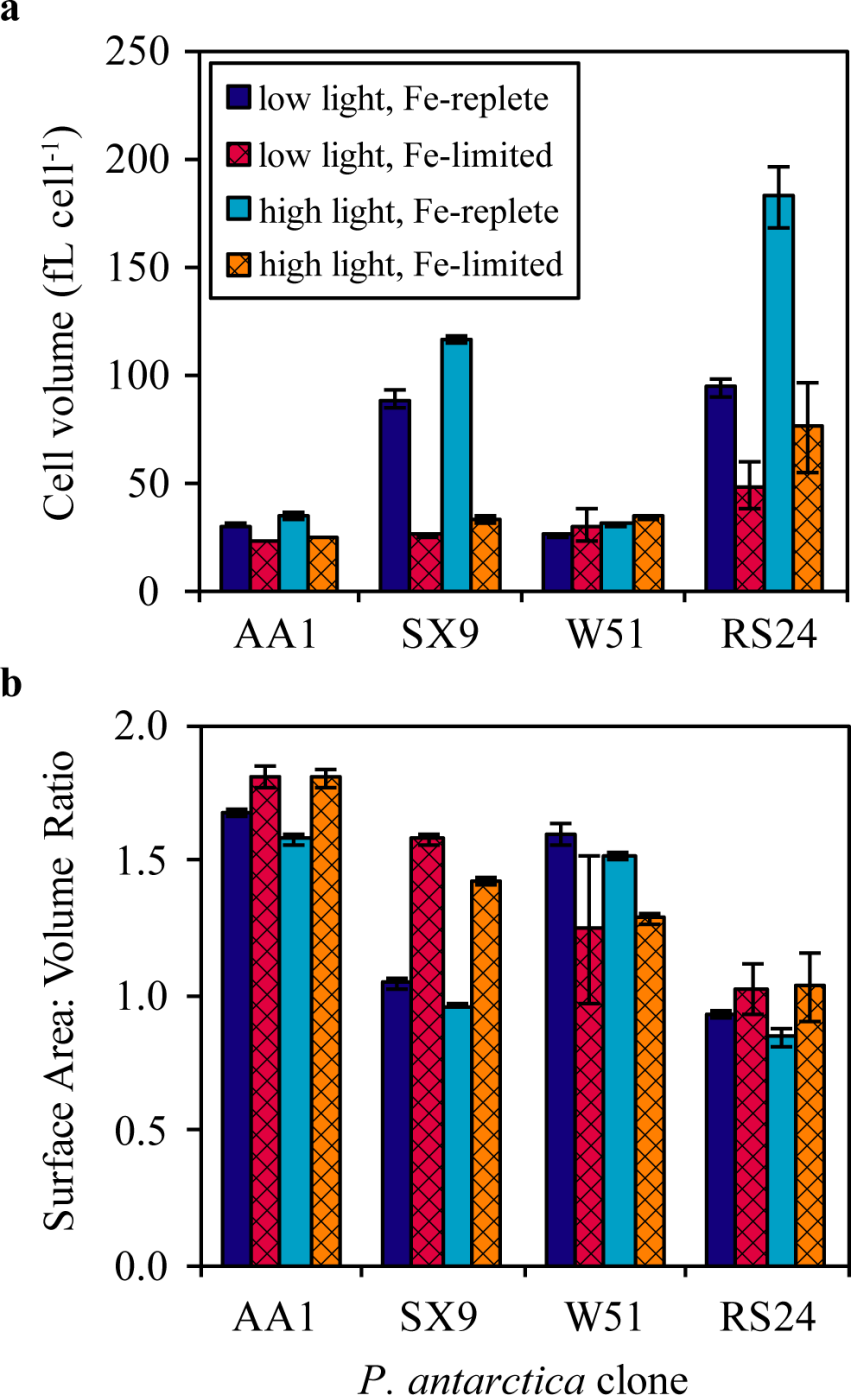
Changes in cell size upon iron limitation vary among the four *P*. *antarctica* clones. These graphs plot the cell volume (**a**) and surface area to volume ratios (**b**) of solitary cells from the four *P*. *antarctica* clones grown under iron limiting and iron replete conditions. The iron limiting conditions, described in the Materials and Methods, were chosen so that each clone had approximately the same degree of iron limitation for these measurements, i.e. half of the iron replete growth rate. The error bars represent the standard error of the mean of three measurements from each of three biological replicates (n = 3). The data are included in S1 and S2 Tables.

Despite a 60-fold difference in Fe' concentration, clone W51 grows comparably in the iron replete and high EDTA media (Table 1). This suggests that the low Fe' concentration in the high EDTA medium is already sufficient to meet the iron demands of clone W51, which may be lower because it is small relative to clones SX9 and RS24 and grows slowly even when iron replete. However, clone W51 does not grow in the high DFB medium. This is surprising because the rate of FeDFB uptake by iron limited eukaryotic phytoplankton is proportional to cellular surface areas [8] and clone W51 is always small (Fig 3, S1 and S2 Tables). Our data hints that clone W51 may lack certain mechanisms to help take up DFB-bound iron, such as Fe(III) concentrating machinery [21] or direct assimilation pathways for the entire complex [22], perhaps because its lower iron requirements decrease selective pressures compared to the other clones.

### Effect of iron limitation on cell size, photosynthetic pigments, and colony formation

*P*. *antarctica* can respond to low iron bioavailability by upregulating its iron uptake machinery, changing its cell size, or decreasing the concentration of iron utilizing enzymes [5]. To determine whether the clones exhibit a similar physiological response to low iron, we grew them at the same relative iron limitation and measured intracellular chlorophyll *a* concentrations, cell size distributions, and colony formation.

Cell size and iron uptake rates are strongly correlated in iron limited phytoplankton [8]. We observed a statistically significant decrease in cell size upon iron limitation in clones SX9, RS24 and AA1 (Fig 3, S1 and S2 Tables), compatible with observations made for *Phaeocystis* spp. in past studies (e.g. [5, 23]). Whereas clones SX9 and RS24 decreased their cell volume by more than half in response to iron limitation, the always small cell volumes of clones W51 and AA1 (<50 fL cell^-1^ even under iron replete conditions) suggest that iron uptake by these clones is already, if inadvertently, benefiting from larger surface area to volume ratios.

**Fig 4:**
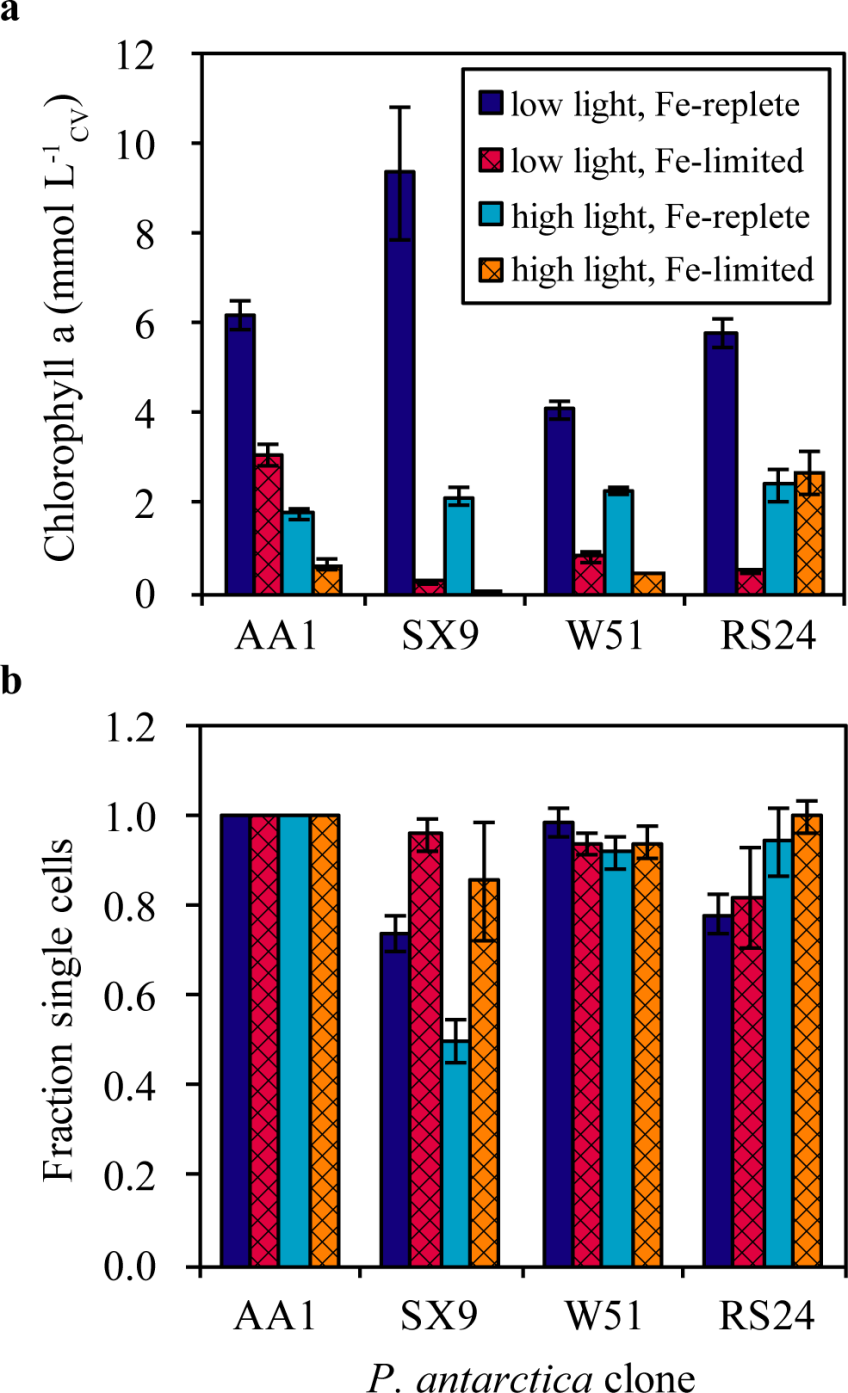
Intracellular chlorophyll *a* concentrations and the fraction of solitary cells in *P*. *antarctica* clones under iron replete and iron limited growth. These plots show (**a**) the intracellular chlorophyll *a* concentrations and (**b**) the fraction of solitary cells (as opposed to colonial cells) of the four *P*. *antarctica* clones grown under different iron- and light-conditions described in the text. The error bars represent the standard error of the mean of three measurements from each of three biological replicates (n = 3), except for clone AA1 in (**b**) where only solitary cells were observed previously by microscopy. The data are shown in S3 (chlorophyll *a* concentrations) and S4 (percent solitary cells) Tables.

Biosynthesis of chlorophyll *a*, an important photosynthetic pigment, involves many Fe-containing proteins [24, 25]. Phytoplankton alter the expression of metabolic pathways associated with iron utilization in response to iron limitation (e.g. [26] [27]). As seen in previous experiments with *P*. *antarctica* [6], intracellular chlorophyll *a* concentrations are significantly (p < 0.05) and substantially (fold-change > 2) lower under iron limitation at both high and low light for clones SX9, AA1 and W51 (Fig 4A, S3 Table). Unexpectedly, for clone RS24 grown at high light, the intracellular chlorophyll *a* concentrations are statistically indistinguishable in the iron limiting and iron replete treatments. Regulating chlorophyll biosynthesis is only one strategy to decrease cellular iron demands and adapt to iron limiting conditions, but it is interesting that the four clones differentially exhibit this regulatory acclimation.

We also measured the fraction of solitary and colonial cells in each culture (Fig 4B, S4 Table). The relationship between nutrient acquisition and colony formation by *P*. *antarctica* remains elusive [1], although field observations (colonial blooms tend to form in the spring, when melting sea ice releases iron) and *in situ* experiments suggest that colony formation is favored by high iron availability [28]. Additionally, in culture, it has been previously observed that clone SX9 forms colonies only when iron replete; clone AA1 lost the ability to form colonies after three years in culture, but only formed colonies under iron replete conditions while it retained this ability [5]. In our experiments, clone SX9 formed significantly more colonial cells when iron replete and at high light intensity. Clone RS24 formed more colonial cells under high light conditions when iron replete, although this difference is not statistically significant. Clones AA1 and W51 formed no or very few colonies under all conditions. Thus, our results are compatible with the hypothesis that colony formation is favored under high light, high iron conditions.

### Clones’ acclimate to iron limitation differently

Our data demonstrate that the four *P*. *antarctica* clones exhibit varying physiological responses to iron limitation and are differentially iron limited under the same external low iron conditions (Table 2, S5 Table). In brief, clone W51 is small, grows slowly, and exhibits chlorosis; clone AA1 is small and exhibits chlorosis; clone SX9 decreases its cell size, forms fewer colonies and exhibits chlorosis in response to iron limitation; and clone RS24 decreases its cell size in response to iron limitation. Clone W51 is not iron limited in our low Fe' treatment, but struggles to grow using DFB-bound iron. Clones AA1, SX9 and RS24 are iron limited in the low Fe' treatment, but grow better than clone W51 using DFB-bound iron. These differences are surprisingly large for isolates of what is considered a single phytoplankton species [29].

**Table 2:** Cultured *P*. *antarctica* clones respond differently to iron limitation.

	Clone			
	AA1	SX9	W51	RS24
Grows in the high DFB treatment	+	+	-	+
Grows well in the high EDTA treatment (low Fe')	+	-	+	-
Cell size depends on Fe status	+	+	-	+
Cell size depends on light conditions	-	+	+	+
Exhibits chlorosis	+	+	+	-
Fewer colonial cells when iron- or light-limited	-	+	-	-

This table summarizes the acclimations observed for each of the four *P*. *antarctica* clones in response to iron limitation. “+” indicates that the strategy was observed in our experiments, whereas “-”indicates it was not. The thresholds used to determine utilization of strategy are shown in the supporting information (S5 Table).

### Environmental relevance

Genetic surveys in the Southern Ocean have suggested that *P*. *antarctica* has different biogeographical ecotypes [30, 31] and it was recently shown that the species’ genetic variability is high over space and time, even among clones isolated from the same *P*. *antarctica* bloom [29]. It is unclear whether physiological gradients follow the same patterns (e.g. [32, 33]). Our study has shown that *P*. *antarctica* clones cultured under identical conditions can exhibit different growth rate patterns and varying abilities to grow using organically bound iron. However, the physiological differences among the four clones studied here do not appear to be explained by the location or time of their isolation (Table 3). Clones W51 and RS24 were isolated from the Ross Sea, whereas clones AA1 and SX9 were isolated from the open ocean, but neither of these pairs respond similarly to low iron. This contrasts previous studies with cultured marine phytoplankton, where physiological differences could be clearly linked to isolation in coastal waters vs. the open ocean [34, 35].

**Table 3:** Information about the *P*. *antarctica* clones used in this study.

Clone	Isolated By	Isolation Date	Isolation Location
AA1	R. Strzepek	Dec-01	61°20.89' S, 139°50.69' E
SX9	R. Strzepek	Dec-04	65°08.72' S, 174°08.94' E
W51	D. Caron	Jan-99	Ross Sea
RS24	D. Caron	Jan-05	Ross Sea
			

Experiments were performed in 2005 (clone AA1) and 2013 (clones SX9, RS24 and W51). Since isolation, the clones studied here have been maintained in culture in an EDTA-based medium. All four clones compared here were isolated from colonies, but clone AA1 stopped forming colonies after ~3 years in culture [5], which may be related to a lack of sexual reproduction [41]. Clone AA1 has been studied repeatedly since its initial isolation, and as reported previously, its growth rate did not change over a five year period under the conditions used here [5]. The original physiology of clones RS24 and W51 is unknown.

Another potential driver of intraspecific diversity is functional redundancy [36]. This has been reported for other phytoplankton species ([37] and references therein) and could be a way for *P*. *antarctica* to thrive in the highly variable conditions in the Southern Ocean (e.g. due to melting sea ice in the spring [38], seasonally variable dust inputs [39], and varying light conditions [1]). It is difficult to assess the role of functional redundancy in *P*. *antarctica*’s success based on our data alone. However, based on the growth rate patterns observed here, we hypothesize that clones like SX9 and RS24 could be better suited for rapid growth as iron availability changes, whereas clones like AA1 and W51 may be able to grow more slowly but steadily across a wider range of environmental conditions. As the *P*. *antarctica* proteome becomes better mapped, a promising strategy to uncover the distribution of physiological *P*. *antarctica* diversity in the Southern Ocean could mimic the approach used by Saito *et al*. to map functional differences with respect to nitrogen acquisition [40].

Our data clearly demonstrate that individual clones within the *P*. *antarctica* species use different strategies to deal with the variably low iron and light conditions in the Southern Ocean. Their physiological diversity results in differing abilities to grow under the same external iron conditions. These results highlight the caveats associated with extrapolating the physiology of a single clone to the entire species, and suggest that future efforts should focus more on understanding the source and distribution of *P*. *antarctica*’s physiological diversity.

## Supporting information

S1 Table Average cell volume of the *P*. *antarctica* clones grown under different iron- and light-conditions. This table provides the average cell volume (fL cell^-1^) measured for each of the four *P*. *antarctica* clones grown under different iron- and light-conditions. Each biological replicate was measured three times, and the standard error of the mean cell volume from three biological replicates is provided (n = 3). These data are plotted in Fig 3A. The data from clone AA1 have been published previously (Strzepek *et al*. 2011 [low light], Strzepek *et al*. 2012 [high light]).
(DOCX)

S2 Table Cell surface area to volume ratios (μm^-1^) of the *P*. *antarctica* clones grown under different iron- and light-conditions. This table provides the cell surface area to volume ratios (μm^-1^) of the four *P*. *antarctica* clones grown under different iron- and light-conditions. Each biological replicate was measured three times, and the standard error of the mean ratio from three biological replicates is provided (n = 3). These data are plotted in Fig 3B. The data from clone AA1 have been published previously (Strzepek *et al*. 2011 [low light], Strzepek *et al*. 2012 [high light]).
(DOCX)

S3 Table Intracellular chlorophyll a concentrations of the four *P*. *antarctica* clones grown under different iron- and light-conditions. This table provides the intracellular cell volume-normalized chlorophyll *a* concentrations (mmol chlorophyll *a* L_CV_^-1^) of the four *P*. *antarctica* clones grown under different iron- and light-conditions. Each biological replicate was measured three times, and the standard error of the mean chlorophyll *a* concentration from three biological replicates is provided (n = 3). These data are plotted in Fig 4A. The data from clone AA1 have been published previously (Strzepek *et al*. 2011 [low light], Strzepek *et al*. 2012 [high light]).
(DOCX)

S4 Table Percentage of solitary cells of the four *P*. *antarctica* clones grown under different iron- and light-conditions. This table provides the percentage of solitary cells (out of the total solitary + colonial cells) of the four *P*. *antarctica* clones grown under different iron- and light-conditions. Each biological replicate was measured three times, and the standard error of the mean percentage from three biological replicates is provided (n = 3). These data are plotted in Fig 4B.
(DOCX)

S5 Table Criteria used to determine whether clones exhibit a particular response to the iron limiting treatments. This table shows the statistical significance (p-value) and fold-change (FC) thresholds which were used to identify clones that exhibit particular responses, such as a change in cell size or growth limitation, to the iron limiting treatments. The results of this analysis are shown in Table 2.
(DOCX)
