# Supplementary material for: Intraspecific variability in Phaeocystis antarctica's response to iron and light stress
Source: PLoS One. 2017 Jul 10;12(7):e0179751. doi: 10.1371/journal.pone.0179751 (PMC5503234; doi:10.1371/journal.pone.0179751)
Supplement: S1 Table — This table provides the average cell volume (fL cell-1) measured for each of the four P. antarctica clones grown under different iron- and light-conditions. Each biological replicate was measured three times, and the standard error of the mean cell volume from three biological replicates is provided (n = 3). These data are plotted in Fig 3A. The data from clone AA1 have been published previously (Strzepek et al. 2011 [low light], Strzepek et al. 2012 [high light]). (DOCX) [file pone.0179751.s001.docx]

**Table S1. Average cell volume of the *P. antarctica* clones grown under different iron- and light-conditions.**

| *mean* |  | **AA1** | **SX9** | **W51** | **RS24** |
| --- | --- | --- | --- | --- | --- |
| **low light** | **Fe replete** | 30 | 89 | 26 | 94 |
|  | **Fe limited** | 23 | 26 | 30 | 48 |
| **high light** | **Fe replete** | 34 | 116 | 31 | 182 |
|  | **Fe limited** | 24 | 33 | 34 | 75 |
|  |  |  |  |  |  |
| *standard error* | | **AA1** | **SX9** | **W51** | **RS24** |
| **low light** | **Fe replete** | 0.6 | 4.9 | 0.7 | 4.1 |
|  | **Fe limited** | 0.4 | 0.9 | 7.6 | 10.9 |
| **high light** | **Fe replete** | 1.5 | 1.7 | 0.7 | 13.8 |
|  | **Fe limited** | 0.5 | 1.6 | 1.2 | 20.6 |

This table provides the average cell volume (fL cell^-1^) measured for each of the four *P. antarctica* clones grown under different iron- and light-conditions. Each biological replicate was measured three times, and the standard error of the mean cell volume from three biological replicates is provided (n = 3). These data are plotted in Fig 3a. The data from clone AA1 have been published previously (Strzepek *et al*. 2011 [low light], Strzepek *et al*. 2012 [high light]).
