# Supplementary material for: Intraspecific variability in Phaeocystis antarctica's response to iron and light stress
Source: PLoS One. 2017 Jul 10;12(7):e0179751. doi: 10.1371/journal.pone.0179751 (PMC5503234; doi:10.1371/journal.pone.0179751)
Supplement: S2 Table — This table provides the cell surface area to volume ratios (μm-1) of the four P. antarctica clones grown under different iron- and light-conditions. Each biological replicate was measured three times, and the standard error of the mean ratio from three biological replicates is provided (n = 3). These data are plotted in Fig 3B. The data from clone AA1 have been published previously (Strzepek et al. 2011 [low light], Strzepek et al. 2012 [high light]). (DOCX) [file pone.0179751.s002.docx]

**Table S2. Cell surface area to volume ratios (µm^-1^) of the *P. antarctica* clones grown under different iron- and light-conditions.**

| *mean* |  | **AA1** | **SX9** | **W51** | **RS24** |
| --- | --- | --- | --- | --- | --- |
| **low light** | **Fe replete** | 1.68 | 1.04 | 1.60 | 0.93 |
|  | **Fe limited** | 1.81 | 1.58 | 1.24 | 1.02 |
| **high light** | **Fe replete** | 1.58 | 0.96 | 1.51 | 0.84 |
|  | **Fe limited** | 1.80 | 1.42 | 1.28 | 1.03 |
|  |  |  |  |  |  |
| *standard error* | | **AA1** | **SX9** | **W51** | **RS24** |
| **low light** | **Fe replete** | 0.01 | 0.02 | 0.04 | 0.01 |
|  | **Fe limited** | 0.04 | 0.02 | 0.28 | 0.09 |
| **high light** | **Fe replete** | 0.02 | 0.01 | 0.01 | 0.03 |
|  | **Fe limited** | 0.03 | 0.02 | 0.02 | 0.12 |

This table provides the cell surface area to volume ratios (µm^-1^) of the four *P. antarctica* clones grown under different iron- and light-conditions. Each biological replicate was measured three times, and the standard error of the mean ratio from three biological replicates is provided (n = 3). These data are plotted in Fig 3b. The data from clone AA1 have been published previously (Strzepek *et al*. 2011 [low light], Strzepek *et al*. 2012 [high light]).
