# Supplementary material for: Intraspecific variability in Phaeocystis antarctica's response to iron and light stress
Source: PLoS One. 2017 Jul 10;12(7):e0179751. doi: 10.1371/journal.pone.0179751 (PMC5503234; doi:10.1371/journal.pone.0179751)
Supplement: S3 Table — This table provides the intracellular cell volume-normalized chlorophyll a concentrations (mmol chlorophyll a LCV-1) of the four P. antarctica clones grown under different iron- and light-conditions. Each biological replicate was measured three times, and the standard error of the mean chlorophyll a concentration from three biological replicates is provided (n = 3). These data are plotted in Fig 4A. The data from clone AA1 have been published previously (Strzepek et al. 2011 [low light], Strzepek et al. 2012 [high light]). (DOCX) [file pone.0179751.s003.docx]

**Table S3. Intracellular chlorophyll a concentrations of the four *P. antarctica* clones grown under different iron- and light-conditions.**

| *mean* |  | **AA1** | **SX9** | **W51** | **RS24** |
| --- | --- | --- | --- | --- | --- |
| **low light** | **Fe replete** | 6.20 | 9.35 | 4.08 | 5.77 |
|  | **Fe limited** | 3.08 | 0.26 | 0.80 | 0.50 |
| **high light** | **Fe replete** | 1.76 | 2.16 | 2.26 | 2.40 |
|  | **Fe limited** | 0.64 | 0.06 | 0.45 | 2.67 |
|  |  |  |  |  |  |
| *standard error* | | **AA1** | **SX9** | **W51** | **RS24** |
| **low light** | **Fe replete** | 0.34 | 1.47 | 0.17 | 0.31 |
|  | **Fe limited** | 0.23 | 0.02 | 0.15 | 0.02 |
| **high light** | **Fe replete** | 0.10 | 0.20 | 0.09 | 0.39 |
|  | **Fe limited** | 0.09 | 0.00 | 0.02 | 0.49 |

This table provides the intracellular cell volume-normalized chlorophyll *a* concentrations (mmol chlorophyll *a* L_CV_^-1^) of the four *P. antarctica* clones grown under different iron- and light-conditions. Each biological replicate was measured three times, and the standard error of the mean chlorophyll *a* concentration from three biological replicates is provided (n = 3). These data are plotted in Fig 4a. The data from clone AA1 have been published previously (Strzepek *et al*. 2011 [low light], Strzepek *et al*. 2012 [high light]).
