# Supplementary material for: Intraspecific variability in Phaeocystis antarctica's response to iron and light stress
Source: PLoS One. 2017 Jul 10;12(7):e0179751. doi: 10.1371/journal.pone.0179751 (PMC5503234; doi:10.1371/journal.pone.0179751)
Supplement: S4 Table — This table provides the percentage of solitary cells (out of the total solitary + colonial cells) of the four P. antarctica clones grown under different iron- and light-conditions. Each biological replicate was measured three times, and the standard error of the mean percentage from three biological replicates is provided (n = 3). These data are plotted in Fig 4B. (DOCX) [file pone.0179751.s004.docx]

**Table S4. Percentage of solitary cells of the four *P. antarctica* clones grown under different iron- and light-conditions.**

| *mean* |  | **AA1** | **SX9** | **W51** | **RS24** |
| --- | --- | --- | --- | --- | --- |
| **low light** | **Fe replete** | 100% | 74% | 99% | 78% |
|  | **Fe limited** | 100% | 96% | 94% | 82% |
| **high light** | **Fe replete** | 100% | 50% | 92% | 94% |
|  | **Fe limited** | 100% | 86% | 94% | 85% |
|  |  |  |  |  |  |
| *standard error* | | **AA1** | **SX9** | **W51** | **RS24** |
| **low light** | **Fe replete** | - | 4% | 3% | 4% |
|  | **Fe limited** | - | 3% | 2% | 11% |
| **high light** | **Fe replete** | - | 5% | 4% | 8% |
|  | **Fe limited** | - | 13% | 3% | 5% |

This table provides the percentage of solitary cells (out of the total solitary + colonial cells) of the four *P. antarctica* clones grown under different iron- and light-conditions. Each biological replicate was measured three times, and the standard error of the mean percentage from three biological replicates is provided (n = 3). These data are plotted in Fig 4b.
