# Supplementary material for: Intraspecific variability in Phaeocystis antarctica's response to iron and light stress
Source: PLoS One. 2017 Jul 10;12(7):e0179751. doi: 10.1371/journal.pone.0179751 (PMC5503234; doi:10.1371/journal.pone.0179751)
Supplement: S5 Table — This table shows the statistical significance (p-value) and fold-change (FC) thresholds which were used to identify clones that exhibit particular responses, such as a change in cell size or growth limitation, to the iron limiting treatments. The results of this analysis are shown in Table 2. (DOCX) [file pone.0179751.s005.docx]

**Table S5. Criteria used to determine whether clones exhibit a particular response to the iron limiting treatments.**

|  | **Criteria** |
| --- | --- |
| Grows in the high DFB treatment | µ > 0 in Fe:DFB 4:400 medium |
| Grows well in the high EDTA treatment (low Fe') | µ:µ_max_ is > 0.85 |
| Cell size depends on Fe status | p < 0.05, FC > 1.3 |
| Cell size depends on light conditions | p < 0.05, FC > 1.3 |
| Exhibits chlorosis | p < 0.05, FC indicating chlorosis |
| Fewer colonial cells when iron- or light-limited | p < 0.05 |

^a^ Clone RS24 (low light) was included as well (p = 0.059, FC = 1.9).

This table shows the statistical significance (p-value) and fold-change (FC) thresholds which were used to identify clones that exhibit particular responses, such as a change in cell size or growth limitation, to the iron limiting treatments. The results of this analysis are shown in Table 2.
